# Diabetic Foot Due to Anaphylactic Shock: A Case Report

**DOI:** 10.5812/atr.17610

**Published:** 2014-06-01

**Authors:** Ali Karakus, Mustafa Ozkan, Murat Karcioglu, Raif Ozden, Ihsan Ustun, Koca Caliskan, Cumali Gokce, Mustafa Sahan

**Affiliations:** 1 Department of Emergency Medicine, Faculty of Medicine, Mustafa Kemal University, Hatay, Turkey; 2 Department of Plastic Surgery, Faculty of Medicine, Mustafa Kemal University, Hatay, Turkey; 3 Department of Anesthesia, Faculty of Medicine, Mustafa Kemal University, Hatay, Turkey; 4 Department of Orthopedic Surgery, Faculty of Medicine, Mustafa Kemal University, Hatay, Turkey; 5 Department of Endocrinology, Faculty of Medicine, Mustafa Kemal University, Hatay, Turkey

**Keywords:** Snake Bite, Anaphylactic Shock, Diabetic Foot

## Abstract

**Introduction:**

Diabetic foot is a clinical disorder, which is commonly seen in patients with diabetes mellitus. It is also the major cause of below knee amputation in the world. There are many underlying causes such as neuropathic, ischemic, and infectious causes for diabetic foot. Local or systemic complications may develop after snake bite.

**Case Presentation:**

We reported a very rare case, involving a 78-year-old male admitted to the Emergency Department, who developed anaphylactic shock and diabetic foot after the snake bite.

**Conclusions:**

Reviewing the literature, this is the second reported case of snake bite associated with diabetic foot.

## 1. Introduction

Local and systemic complications can develop as a result of snake bite (SB). Ulcers at bite site, anaphylactic reactions, respiratory and cardiac adverse events and death may be seen. Diabetic foot (DF) ulcers are the lesions which can develop due to various reasons, and cause complications in these patients. The most common type is neuropathic ulcers, which develop after trauma (1).

 Anaphylaxis is a severe allergic reaction caused by release of inflammatory mediators from mast cells and basophile via immunoglobulin E (IgE)-mediated pathway that may involve organs too. In this article, we report a very rare case of a patient who developed anaphylactic shock and DF after the SB. To the best of our knowledge, this is, the second case of SB associated with DF, which has been reported in the literature.

## 2. Case Presentation

A 78-year-old man was admitted to the State Hospital with an SB (by a viper) on his left leg one hour earlier. The patient was moderately conscious during the admission. His vital signs were as follows: blood pressure, 80/40 mmHg; pulse, 120 beats per minute; respiratory rate, 25 breaths per minute; and body temperature, 36.5°C. His skin was faint and sweaty; there was a bite at the lateral aspect of the tibia in left leg and edema of left foot.

 The patient had a history of 20 years of diabetes mellitus (DM) treated with insulin for 10 years. He also had diagnosis of prostate cancer for one year.

 In the initial laboratory evaluations, the following results (normal range) were found: white blood cell, 12.1 × 10³ µ/L (4.6-10.2); glucose, 181 mg/dL (70-105) (to convert to mmol/L, multiply by 0.055); blood urea nitrogen, 39 mg/dL (7–26); prothrombin time (PT), 16.2 s (10-15); pH = 7.31 (7.35-7.45): HCO_3_, 24 mmol/L (24-28): PCO_2_, 29 mmHg (35-45) (to convert to kPa, multiply by 0.133): SO_2_, 93 mmHg (95-100).

Electrocardiography (ECG), showed a sinus arrhythmia, whereas, in echocardiography, there were satisfactory systolic functions, as well as minimal pulmonary hypertension. The patient developed respiratory distress and anaphylactic reaction within an hour after the hospitalization.

 Intramuscular adrenalin 0.5 mg was administered with an initial diagnosis of anaphylactic reaction, and the patient admitted to intensive care unit, with the following medications: antivenom infusion (4 vials in 100 mL normal saline, over 1 hour); insulin infusion; 1 g cefazoline sodium (three times in a day); and 300 mg acetyl salicylic acid.

Up to the clinical improvement, antivenom therapy was maintained, and totally; 16 antivenom vials were given. Dopamine infusion (10 µg/kg/min) was also given to the patient to treat his hypotension. Diuretic therapy was initiated due to hearing crackles in the lung auscultation.

 In the control blood gas analysis, pH, HCO_3_ and lactate were found as 7.01, 18 and 12, respectively. Fresh frozen plasma (2 units) was given to the patient because of the prolonged international normalized ratio (INR).

**Figure 1.**
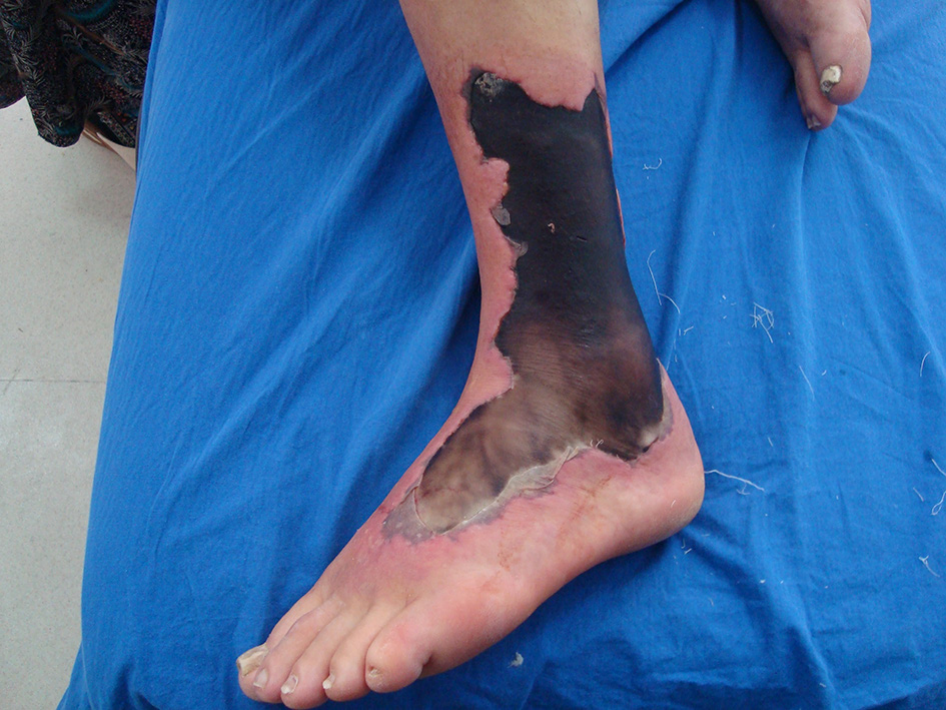
Diabetic Foot and Necrotic Areas After Snake Bite

 Necrotic tissue (5 × 10 cm in size) developed at the lateral aspect of his involved leg 2 days later (Figure 1). In the microscopic evaluation of the wound sample, abundant erythrocytes and scarce leukocytes were detected. However, no bacterium was found.

**Figure 2.**
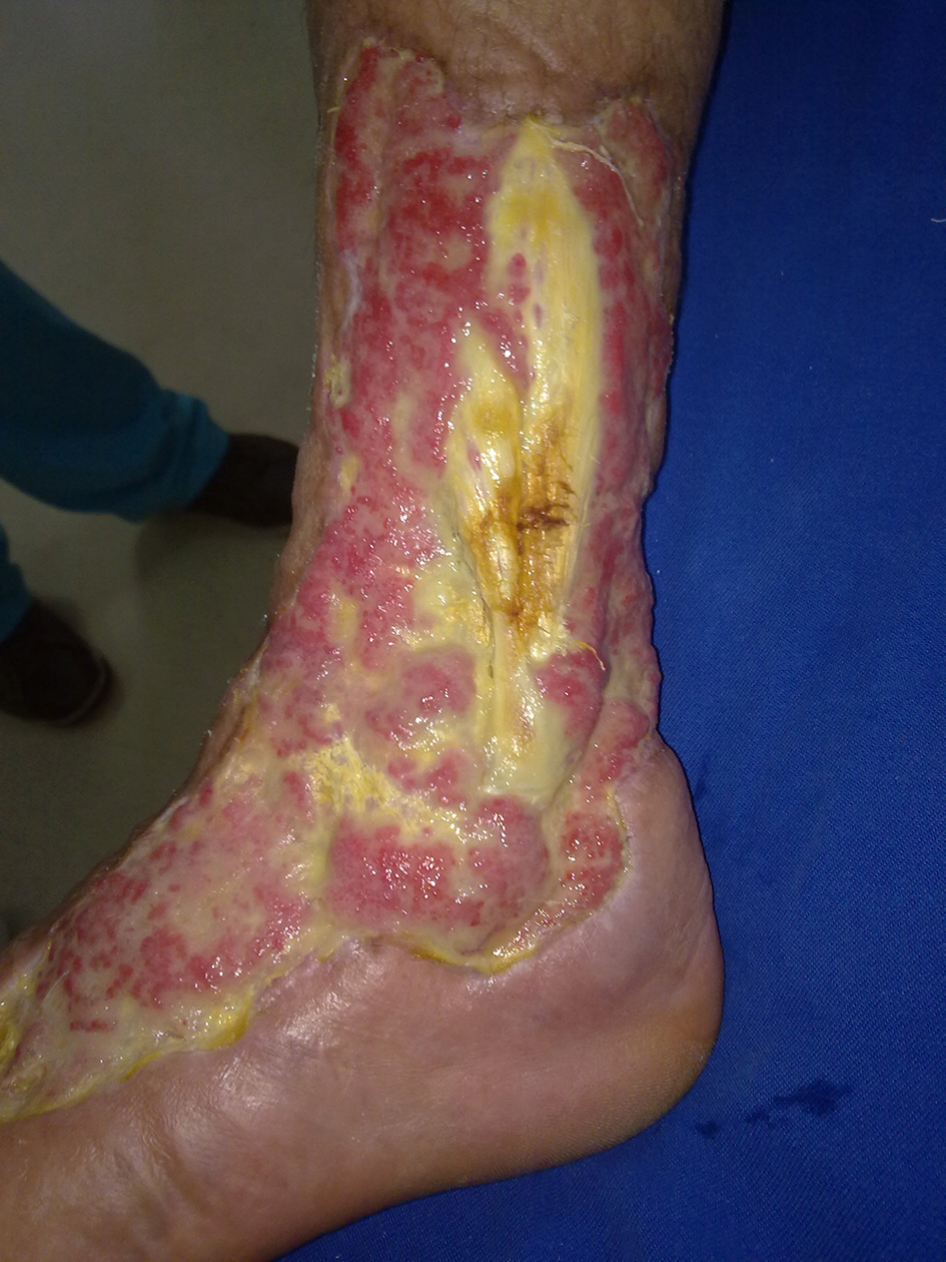
After Debridement and Grafting

 In the arterial Doppler sonography of lower extremities, calcified atherosclerotic changes were observed at the arterial system. Debridement and a partial thickness skin graft were performed by a plastic surgeon. Mesh treatment was scheduled, but the patient refused this treatment, and discharged (Figure 2).

## 3. Discussion

DF is a clinical disorder, which is commonly seen in patients with DM. It is also the major cause of below knee amputation in the world. There are many underlying causes such as neuropathic, ischemic and infectious causes for Diabetic foot (2). The risk of developing foot ulcer is 15% in patients with DM, which is accounted for half of the nontraumatic amputations (1). The Wagner-Megitt classification which is used in DF, has five grades (0-5) based on the depth of ulcer and extent of gangrene (Table 1) (3).

**Table 1.** Wagner-Megitt Classification

Grades	Results
**Grade 0**	no foot ulcers
**Grade 1**	superficial ulcers
**Grade 2**	deep ulcer (cellulitis)
**Grade 3**	ulceration, osteomyelitis, or abscess
**Grade 4**	the formation of local gangrene
**Grade 5**	diffuse gangrene

Snake venom comprises histamine and histamine-like substances in addition to proteolytic, and hydrolytic enzymes, as well as hyaluronidase. Local tissue injury is caused by myotoxic and cytolytic effects of the toxin (4). As a result, gas gangrene and soft tissue infection may develop, which are caused by *Clostridium* spp. or *Aeromonas hydrophila* (5).

 In a study regarding the etiology of chronic skin ulcers, incidence of diabetic ulcers was found 4.91%. However, the ulcers caused by SB were classified under the other etiologies (6). In another study, it was reported that skin necrosis related to SB developed in 10% of the cases of chronic skin ulcers (7). In the literature, only one case with DM developed local cellulitis after SB (8). As it is mentioned in the literature, soft tissue problems are usually seen in diabetic patients.

 However, the interesting point of our case was that the patient had not only diabetes mellitus but also a snake bite before the occurrence of diabetic foot. Anaphylactic reaction may develop due to IgE-related mediators after SB (7). Allergen, IgE pathway, mediators released from mast cell and basophils are all involved in this reaction. The causes of anaphylactic reaction include drugs, antibiotics, transfusion of blood products and environmental factors such as SB. It may affect skin, gastrointestinal system, respiratory system, cardiovascular system and nervous system. Flushing, itching, urticaria, angioedema and bronchospasm, shortness of breath, chest pain, seizure, respiratory arrest, cardiac arrhythmia, hypotension, shock and death may be seen.

 In case of anaphylaxis, basic life support should be provided. Oxygen, adrenalin diphenhydramine, prednisolone, fluid and positive inotropic agents, if indicated, may be needed. In our case, systemic involvement was seen after the local findings of anaphylaxis. Clinical improvement was seen after appropriate management. Snake venom may cause local and systemic symptoms and signs due to its neurotoxin, cytotoxin and hematotoxin. The most commonly seen symptoms and signs are bite site, swelling, and flushing (9). The patient may also develop nausea, vomiting, respiratory distress, palpitation, and loss of consciousness (5).

 Proteins in the venom toxin may affect all the systems by causing endothelial and plasma membrane disorder. Ischemic injury and tissue edema occur due to the effects of the toxin on tissues (10). DM patients who were bitten are prone to acute DM complications like comatose state (diabetic ketoacidosis, hyperglycemic hyperosmolar state) and chronic DM complications such as nephropathy, and peripheral vascular complications, which worsen snake bite ulcer and its complications.

Extravasation of the electrolytes and erythrocytes occur because of increased capillary permeability, hemolysis, edema, hypovolemic shock and lactic acidosis. Especially in older patients, systemic complications can be developed, which may increase mortality. Therefore, elderly patients should be cautious in the clinical follow-up, and vital signs should be monitored frequently. In the present case, there were local and systemic findings as mentioned in the report.

In the SB, the first goal is to provide basic life support, if needed, followed by other supportive measures. Antivenom therapy, the primary treatment in SB, is recommended via intravenous route, particularly in patients with progressive symptoms, abnormal ECG pattern, loss of consciousness, hypotension, shock, coagulopathy, compartment syndrome, and renal failure (11, 12). Compartment syndrome develops because of muscular necrosis rather than vascular failure.

 Additional antivenom therapy should be given in patients with no improvement in clinical findings, compartment syndrome and coagulopathy. If hypotension persists, inotropic agents can be considered, whereas appropriate blood replacement in coagulopathy and fasciotomy in the compartment syndrome. Amoxicillin clavulanate combination or ceftriaxone is preferred in order to target *Clostridium* and *Proteus* species transferred from oral flora of the snake to the wound. In our case, antivenom therapy was initiated due to development of abnormal ECG pattern, loss of consciousness, hypotension, shock, and coagulopathy.

 In conclusion, in the SB, there is a risk for the development of DF in patients with DM in addition to systemic effects, including anaphylaxis and cardiac involvement. Particularly, elderly patients should be appropriately managed for these complications. DF may result in foot amputation if regular and good care and management of the wound are not provided.

### 3.1. Supplement

This case was presented in European Society for Emergency Medicine (EuSEM), European Congress on Emergency Medicine, Emergency Physicians Association of Turkey (EPAT) and National Emergency Medicine Congress Antalya, Turkey, 3-6 October 2012.
